# Recruitment and Lessons Learned from a Community-Based Intervention Program: The Learning Families Project in Hong Kong

**DOI:** 10.3389/fpubh.2018.00016

**Published:** 2018-02-01

**Authors:** Joanna T. W. Chu, Alice Wan, Sunita M. Stewart, Kwok Tung Ng, Tai Hing Lam, Sophia S. Chan

**Affiliations:** ^1^School of Population Health, National Institute for Health Innovation, The University of Auckland, Auckland, New Zealand; ^2^School of Public Health, The University of Hong Kong, Hong Kong, Hong Kong; ^3^Department of Psychiatry, The University of Texas Southwestern Medical Center at Dallas, Dallas, TX, United States; ^4^Christian Family Service Centre, Hong Kong, Hong Kong; ^5^School of Nursing, The University of Hong Kong, Hong Kong, Hong Kong

**Keywords:** recruitment, community-based research, family programs, lessons learned, brief intervention

## Abstract

**Background:**

Recruitment is central to any research project, and recruitment itself should be well documented and researched. We describe our recruitment efforts for a community-based research project—entitled the Learning Families Project—conducted in Hong Kong.

**Methods:**

In collaboration with community stakeholders, residents from a public housing estate were recruited to participate in family programs aimed at enhancing family well-being. Various recruitment strategies were employed including the distribution of 19,200 leaflets, 688 posters, a banner, a kick-off ceremony, 10 promotion activities, 1,000 direct calls, word of mouth, 51 mobile counters, and 10 door-to-door visits. Drawing on field notes, research logs, short questionnaires, and focus group conducted with our community partners and residents, we describe and discuss our recruitment strategies, challenges, and lessons learned.

**Results:**

Over a 9-month period, 980 participants were recruited and participated in our study, exceeding our recruitment goal (860 participants). Several observations were made including active recruitment strategies (i.e., door-to-door and mobile counter) being more effective than passive strategies (i.e., posters and leaflets); the importance of raising project awareness to facilitate recruitment; and the challenges encountered (i.e., burn-out and loss of motivation of staff, decreased community capacity in collaborating in research projects).

**Conclusion:**

The lessons learned include the importance of engaging Chinese communities, utilizing a positive outreach approach, and setting realistic expectations. Although similar recruitment strategies have been reported the West, a number of cultural differences should be taken into account when working with Chinese population. Further research is needed to examine the effectiveness of tailoring recruitment strategies to various populations.

## Introduction

The aim of this article is to report our recruitment experience on a community-based research project, designed to address the concerning rates of family problems in a deprived district in Hong Kong, China. The literature on recruitment for family programs conducted in clinical and non-clinical settings documents substantial challenges (1, 2). Generally, the dissemination of preventive interventions in a given target population is less than 1% (3). Difference in recruitment success may lie with the study topics, recruiters, and/or recruitment strategies (4).

Yancey et al. (5) reported that when eligibility criteria were general, passive methods (i.e., mass media, mailing) were more likely to reach the targeted population, at least for middle and higher socioeconomic status, whereas active methods (i.e., face-to face contact) tended to be more effective when eligibility criteria were very specific. Similarly, Park and Sha (4) found that the use of mass media (i.e., newspaper advertisements) was most efficient in reaching a large number of Asian immigrants in the United States. Use of multiple-phase processes, community meetings, and active stakeholder involvement have been proposed as key strategies for recruitment, particularly for community-based projects (5, 6). Gorelick et al. (7) reported the need of a community network for planning access to community groups. They proposed establishing a community advisory board, appointing a community service coordinator, and organizing a network that includes local members to create public awareness about the study through community organizations, media, and mail.

## Background

While strategies and approaches on how to recruit are accumulating, reported experience outside of Western countries is rare (8, 9). There is a dearth of literature documenting recruitment efforts in Asian populations. Of those that have been reported, most have concentrated on Asian individuals living in Western societies and/or as a minority of the population (4, 10). Engaging individuals in family programs in the East is critical in the process of building knowledge about effective services to Asian and other populations. We describe our recruitment efforts from our community-based project conducted in Hong Kong—entitled the Learning Families Project. We do not intend to evaluate comprehensively the strategies of recruitment, but rather to systematically document the process and the lessons learned. We aim to provide insight and direction for researchers looking to recruit and work in community settings, particularly with Chinese populations. We provide a brief overview of the Learning Families Project, describe our recruitment process, based on field notes, research logs, short questionnaires, and community partners’ and residents’ focus group data, and discuss the lessons learned.

## Description of the Case—The Learning Families Project

The Learning Families Project was part of the project entitled “FAMILY: a Jockey Club Initiative for a Harmonious Society” with the mandate to promote family health, happiness, and harmony in Hong Kong families. Kwun Tong district is a relatively traditional and less privileged community in Hong Kong, with a population of 622,152 (11). Approximately 56% of residents live in public housing estates, approximately 30% are aged 55 years or above, 45.3% have education at or below the elementary school level, with average household income less than HKD$10,000 [34.6%; average household income in Hong Kong was $20,200; US$1 = HKD$7.8 (11)]. The district has high prevalence of elderly abuse (8.2%), domestic violence (9.4%), and child abuse (9.8%) compared with other districts in Hong Kong (12). To address these concerns, the FAMILY Project Team collaborated with the Christian Family Service Centre (CFSC), a non-government organization with its headquarters based in Kwun Tong, and developed the Learning Families Project. The project was conducted at the community level, preventative in nature, between July 2010 and March 2012. The overall aim is to improve family well-being of community residents and establish a platform for enhancing social network and neighborhood support in the targeted public housing estate.

One public housing estate was selected as the targeted intervention estate after discussion among HKU staff, CFSC project staff, and various local leaders. The estate consisted of 7 housing blocks with approximately 5,000 households. Families who were Hong Kong residents, able to communicate in Cantonese, and residing in the targeted estate were invited to participate. A feasible and realistic sample size of 860 participants was set upon discussion and agreement with our community partners, steering committee, and funder. Our sample was a convenience sample based on community leaders’ understanding of the community. Ethics approval was obtained from the Institutional Review Board of the University of Hong Kong/Hospital Authority Hong Kong West Cluster.

The objectives of the project are as follows: (1) to promote family well-being of community residents through a series of community-based family programs; (2) to utilize community resources by building capacity among public housing estate resident leaders; and (3) to establish the platform for enhancing social networks and neighborhood support through a series of intervention strategies organized collaboratively by resident leaders, community partners, and academic staff.

The project was conducted in five phases including (1) a needs assessment to determine the emerging issues and concerns in the targeted public housing estate as a basis for capacity-building and intervention planning; (2) capacity-building train-the-trainer programs to engage and equip resident leaders with various skills to design and implement community-based family programs; (3) a series of promotion activities including a kick-off ceremony to raise residents’ awareness of the project; (4) family programs aimed at improving family well-being of residents; and (5) a recognition ceremony to recognize the efforts of community members and for dissemination of findings. The focus of this article is on the planning and development of our recruitment efforts to recruit community residents into the community-based family programs. The outcomes and effectiveness of the project are presented elsewhere (13).

## Methods

### Community as Resources

Our work was guided by a belief in the importance of engaging and creating partnership with representatives of the population to be studied. Community engagement can create a sense of ownership in the local community (14). It can ensure community members make an essential contribution to project development and implementation, addressing concerns and priorities of the community. Building upon relationships with key stakeholders in the targeted district from our previous projects, we formed partnerships and developed a steering committee to oversee and advise the project. The steering committee was responsible for selecting the community/estate that would be part of the study, operationalizing the conceptual framework underlying the project and intervention strategies, helping to define the study population, and, deciding upon recruitment strategies.

### The Unique Role of Resident Leaders

The steering committee believed that resident leaders of the targeted public housing estate would be integral to various phases of the project. The strategy was to engage resident leaders’ in planning and designing family programs and recruiting and delivering family programs to community residents, with support from CFSC staff. As resident leaders were residents of the estate, they were well situated to provide insights about issues and needs of their community. Resident leaders were also more likely to increase community involvement as they provided a readily available platform with strong networks and ties with residents.

A qualitative needs assessment was first conducted with 16 resident leaders. This step was particularly important as it laid the groundwork for community participation and set the stage for collaboration between the research team and resident leaders. By providing the resident leaders an opportunity to voice their opinions, we could foster a personal commitment and their sense of ownership to the project. Further to the needs assessment, we held informal meetings with resident leaders to introduce the project and invited them to collaborate. To ensure buy-in of the project, project staff stressed the significance of research endeavors and potential family and community improvements. Capacity-building train-the-trainer programs were further organized by academic staff and social workers (the typical frontline service delivery professionals in Hong Kong community service agencies) from CFSC to equip resident leaders with relevant knowledge and skills for translating the project aims into effective family programs.

### Developing a Recruitment Plan for Recruiting Community Residents

A recruitment plan was developed during February and March 2011. We worked closely with our community partners to develop, and to modify as needed, the recruitment methods used. Potential barriers (e.g., budget constraints, staff turn-over, and residents’ mistrust in research) and strategies to facilitate recruitment (e.g., local recruiters, ease of access of family programs, souvenirs as incentives) were also identified. Subsequent to initial meetings, phone calls, and small group meetings took place, as necessary. Input from the CFSC project staff and resident leaders helped determine which community members/organizations to contact for recruitment and the best methods for reaching these individuals/organizations. Members of the steering committee also helped researchers to better understand local politics, which often shaped who to contact and how. Resident leaders stressed the need for CFSC and academic staff to become familiar faces in the community as we communicated the benefits of research for the community. Field visits were conducted by the academic and CFSC staff along with resident leaders to understand the dynamics and cues relating to appropriate and sensitive communication and establishing visibility in the community.

Recruitment materials (e.g., posters and leaflets) were developed collaboratively. Specific attention was paid to designing recruitment materials relevant and acceptable to the residents. Simple Chinese language was used to describe the project as part of a larger city-wide effort that was well established, offering family programs that were free of charge, and the benefits of participating.

### Recruitment Strategies

Fieldwork recruitment of residents took place from March to December 2011. A diverse array of recruitment strategies were used concurrently and are described below. Resident leaders, CFSC project staff, and academic staff were actively involved in the recruitment process.

#### Posters, Leaflets, and Banner

Approximately 19,200 leaflets were distributed into mailboxes of the entire estate, recreational centers, and local and community organizations, and at community events at various time during recruitment. In addition, 688 posters were displayed in various community organizations, housing blocks, community events, and Housing Authority office. One banner was also displayed in a conspicuous area in the estate. Community organizations and individuals were encouraged to forward or display information to others who might be interested.

#### Kick-Off Ceremony

To raise awareness of the project, a kick-off ceremony was launched in March 2011 at a common area (i.e., basketball court) within the estate. The aims of the kick-off ceremony are to (1) promote a sense of project ownership among community partners and (2) increase the visibility of the project and the research team to the community residents. Residents were notified of the event *via* posters, word of mouth, and a banner set up within the estate. All of the members of the steering committee attended along with representative from the funding source, Housing Authority, District Council, Social Welfare Department, and a Hong Kong Television celebrity. Stage performances and game booths were hosted by CFSC project staff, resident leaders, and volunteers (of whom a majority were local residents). Logo-adorned items that carried the project name and leaflets with information about the family programs were distributed.

#### Promotion Activities

A total of 10 promotion activities were organized by CFSC staff and resident leaders from April to July 2011 to raise awareness, disseminate information about upcoming family programs, and recruit residents to participate in our family programs. The promotion activities included a range of fun family activities (e.g., Easter egg painting and DIY photo frame). Activities were held in the basketball court of the estate for ease of access. Each activity lasted for 1.5–3 hours and were flexible in delivery allowing residents to come and go. The aims of the promotion activities are to promote and increase the visibility of the project, to form relationships with residents, to become familiar faces in the estate area, and to create a fun and positive environment. Residents were invited to provide their contact details to receive information about our upcoming family programs.

#### Direct Calls/Word of Mouth

Utilizing social networks of CFSC staff, active outreach was conducted by direct contact of potential participants. Approximately 1,000 telephone calls were made to potential participants. Word of mouth was also utilized by CFSC staff, resident leaders, and volunteers to recruit residents and local community organizations to spread the recruitment message. Residents who participated were also encouraged to share and inform others who might be interested, serving as agents to expand recruitment.

#### Mobile Counters

Fifty-one mobile counters were set up within the estate at various times during recruitment to recruit and raise residents’ awareness of the project. To increase residents’ interest, a free health checkup was set up and managed by nursing students from the University of Hong Kong. Leaflets were distributed, and staff made face-to-face contact with passerby residents to introduce the project and recruit residents. These mobile counters similar to the other promotion activities gave community residents insight into the aims of the project and personal contact with the project staff. Daytime and evening, weekday, and weekend slots were necessary to access diverse type of the estate residents.

#### Door-to-Door Canvassing

To reach the hard-to-reach residents, door-to-door visits were conducted. A total of 10 visits reaching approximately 1,000 households were made at various time. For each visit, a total of four staff members including CFSC project staff, resident leaders, and volunteers approached the residents by knocking on the door of each apartment unit during weeknights and weekends. The team introduced the project aims and invited residents to participate in the family programs. Logo-adorned items (e.g., chopsticks, shopping bags) that carried the study name were distributed as incentives to increase interest.

## Results

### Recruitment Success and Challenges

Over a 9-month period, we successfully recruited 980 participants to participate in our family programs, meeting our recruitment goal (860 participants). As we did not obtain data on the numbers of participant recruited *via* each strategy (which is explained below as one of the challenge we encountered), it was difficult to quantify successful enrollment for each strategy or to make direct comparison of different strategies. However, we are able to share our experiences, observations, and challenges based on field notes, research logs, observations, short questionnaires by community residents, and focus groups conducted with our community partners and residents, which are detailed below.

### Passive versus Active Strategies

Based on project staff’ and resident leaders’ feedbacks, active recruitment strategies (i.e., project staff directly contacting prospective participants) were more effective than passive strategies. The use of posters, leaflets, and banner were likely to have reached a diverse population; however, it was difficult to discern how many residents actually received the message. More specifically, how many residents viewed the poster or leaflets could not be determined. This passive form of recruitment that requires prospective participants to contact project staff were described as being “distant” by frontline workers. This was further confirmed in the residents’ focus groups with many reported seeing the posters and leaflets but paid little or no attention to such materials. Nonetheless, the use of posters and leaflets was likely to have contributed to raising awareness and heightening the profile of the project, thereby adding a sense of legitimacy when residents were later approached through other more active recruitment methods. This finding is particularly important as studies conducted in the West have reported that mass media (e.g., newspapers, flyers, and posters) can reach a large group of potential participants and worked particularly well for the general population, especially white participants (4, 15). However, this was not observed in our study. It may be that Chinese individuals are less responsive to passive strategies because of distrust in advertising materials or that they are less willing to make the initial contact with program staff.

In contrast, active strategies (e.g., door-to-door visits, direct call, and mobile counter) were perceived by project staff to be more effective. As stated by a CFSC staff member, door-to-door visit was particularly useful for reaching those who were less active in the community and more difficult to reach. Although some households seemed “cold” and distant at first, learning that the project was a collaboration with resident leaders and local organizations helped break the ice and frontline workers were able to increase residents’ interest in the family programs. These strategies relied heavily on the personal relationships and networks our community partners had with the targeted residents. Frontline workers reported that by explaining directly how participants’ participation would impact those in their estate and how their involvement would benefit them, encouraged residents to participate. However, it was also noted that these strategies were time consuming and labor intensive.

### Raising Awareness of Project

As part of our recruitment process, raising community awareness was an important strategy (e.g., kick-off ceremony and a series of promotion activities). Frontline workers were able to form new and build upon existing relationships and bring about a fun and positive atmosphere of the project into the estate. Based on observations by CFSC staff and academic staff, it was estimated that approximately 1,000 local residents attended or joined parts of the kick-off ceremony. The event was covered by six major mass media outlets. Although a kick-off ceremony may be an uncommon practice in projects in the West, it is a common Hong Kong practice and an important event for our Chinese community partners. The ceremony was supported by the government (i.e., Housing Authority, Social Welfare Department) with officiating guests endorsing the project and encouraging residents’ participation. It was also an event that allowed community partners to support each other and be recognized for their efforts. Based on the Chinese saying that “A good start is half way to success,” the kick-off ceremony symbolized the beginning of a meaningful and successful project. This was also noted by resident leaders in which they perceived the ceremony as successful, attracting many residents, and was therefore an important strategy to raise community awareness and increase community participation.

For the promotion activities, a total of 670 residents participated with 302 residents providing contact information and expressing interest in our family programs. Of the 670 residents, 424 completed a short questionnaire to identify the reasons for participating in the promotion activities. One of the main reasons was that the promotion activities were free. We also found that learning hands-on activities received more positive feedback than seminars or talks. These findings were later used to fine-tune our family programs. Although labor intensive, these promotion activities were well received by both residents and project staff. Resident leaders noted that these activities provided an opportunity for residents to form new relationships with others and be more involved in the community. These newly formed social relationships was confirmed by residents as a motive to participate in subsequent family programs.

### Timing of Recruitment

Our project discovered that timing was an important factor influencing the success of recruitment. For specific strategies, project staff observed that certain time of the day was better suited for recruitment (e.g., weekday night time, weekend before noon for door-to-door visit, between 6 p.m. and 9 p.m. weekdays for using direct calls to potential residents) reaching out to a larger number of potential participants. Similarly, certain months (e.g., May) of the year also appeared to yield higher response than other months. We speculate that this was due to the celebration of Mother’s Day, which reminded residents about the importance of family, and thus, residents were more willing to agree to participate. On the other hand, recruitment in months that were associated with school exams had less response from residents. This was likely because parents were heavily involved in children’s school work and were too busy to participate.

### Burn-Out and Loss of Motivation

Similar to other studies (16), one of the challenges that we encountered was the burn-out and loss of motivation of recruitment staff. Although fieldwork recruitment was conducted over 9 months, the planning and development of a recruitment infrastructure took over half a year. The development of the recruitment plan entailed significant workloads on all parties, including arranging meetings, keeping meeting logs, preparing plain language reports for communities, and progress reports to funding bodies. Moreover, several recruitment strategies were labor intensive (e.g., direct calls, promotion activities, and door-to-door canvassing). Project staff and resident leaders experienced various frustration (e.g., turn-over of staff, slow recruitment, recruitment competition with other organizations/projects, and heavy workload). Even when several local organizations were keen to assist, they had limited resources to allow staff to fully commit their time to active recruitment. The decline of motivation in recruitment and the overall project as the family programs started to roll out was evident. A few resident leaders noted that they underestimated the demand of the project, and the time and effort required to recruit far exceeded their initial expectation. Time off and rotation of recruitment activities were partial responses to potential burn-out and decreased motivation. Additional volunteers were recruited to meet the project’s recruitment demand.

### Community Capacity

Resident leaders and volunteers who had no previous experience with coordinating and managing recruitment, data collection, and issues around scientific validity posed a significant challenge on the project. For example, CFSC project staff, resident leaders, and volunteers were trained (through capacity-building trained-the trainer program, small group meetings, or onsite briefing) to participate in data collection (e.g., keeping logs, field notes, evaluation forms on recruitment, and gathering data from residents *via* questionnaires) either independently or alongside academic staff. However, the capacity-building training program was not sufficient to equip resident leaders with research skills. As a result, we had a substantial number of missing or incomplete forms and data on recruitment (e.g., the numbers of participants recruited *via* each strategy, hours spent on a particular strategy). Questions were also constantly raised by resident leaders and volunteers in terms of why fidelity or evaluation forms had to be completed. As reflected in the resident leaders’ focus group, comments were made in which at times they felt as if they were the research subjects rather than key partners of the project. Some resident leaders and volunteers became impatient at spending time on documenting process rather than on task activities. Given recruitment was not a primary evaluation outcome at the time, we did not aim at complete data collection for recruitment strategies, instead (1) we focused on the data collection on the effectiveness of the family programs and the overall impact of the project; (2) we wanted to reduce the workload of frontline workers; and (3) we did not want the issue to become a source of tension between academic staff and community partners.

## Discussion and Lessons Learned

This study aimed to go beyond the usual social service provisions of interventions to their regular clientele by engaging a number of community partners and extending the reach of family interventions to the whole community. In addition to the observations and challenges noted above, there were several lessons learned on the overall planning and process of recruitment.

### Engaging Chinese Community

Chinese individuals are socially and culturally embedded in their broader Chinese community (17), working with the broader community to recruit and promote our project was essential. With the experiences and expertise in facilitating programs and the established relationships in the estate, our community partners played a key role in the recruitment process. For example, the trust and familiarity that the resident leaders had in the target estate enhanced resident recruitment. This form of partnership between researchers and communities, with community participation contributing to the success of recruitment and community programs, has been described in the literature (14). To facilitate successful recruitment, building relationships and cultivating positive connections in the Chinese community is important. Chinese culture places great emphasis on respectfulness and honor, both of which remain core values of Confucianism (17). We learned that constantly showing appreciation and acknowledging community partners’ effort greatly contributed to the partnership’s ability to establish a strong working relationship. This is reflected in our long-lasting relationships with various community partners who have served as the foundation for other ongoing projects.

### Positive Outreach Approach

It was particularly important to frame the project as a preventive and strengthening approach to support all families. Family programs are often seen as intervention for struggling or “failed” families (18). Seeking support from outside of the family network has been associated by parents with embarrassment or shame, as it can be seen as a sign that the family is “failing” (19). This is particularly relevant to Chinese families, as expressed in a Chinese saying “family shame should not be spread out.” By using a preventive and strengthening approach, we normalized our project, and the family programs were promoted as a source of support for all families and therefore increased family engagement with no stigmatization.

### Setting Realistic Expectations

Recognizing that recruitment is difficult is needed, and often there is an under-estimation of the effort required. Community partners are faced with competing demands, and it is unrealistic to expect community partners to devote all their time and effort into recruitment, particularly for participants beyond their usual service targets. Recruiting people that are hard-to reach is harder than expected but our community partners were willing to try and venture outside their usual comfort zone to expand their service population to benefit more families. Therefore, it is essential to set realistic targets and expectations with community partners to establish respective expectations and responsibilities for a project, taking into account of the changes in personnel, agendas, and budget constraints. Negotiation and discussion are vital in the process. It would be naive and unwise for researchers to expect their enthusiasm would be shared by all partners and the whole targeted community. Having a high degree of flexibility and adaptability to different circumstances is thus necessary. Experiences and lessons learned from the present project also benefited the community partners, organizations, and staff involved.

## Limitations and Conclusion

Given the diversity within Asian ethnic groups, whether our experiences could be generalized is uncertain. However, improving understanding in one population may help raise awareness of possible recruitment issues for other community-based research. Although a range of recruitment strategies were used in our project, the Internet, social networking sites, and social media (e.g., Facebook and Instant messaging) were purposely not used, given the demographic characteristics of the targeted population (i.e., high proportion of people aged 55 years or above) and the uncommon use of social media for recruitment at the time.

It was also difficult to quantify successful enrollment for each strategy separately as they were not independent (although the overall recruitment goal was reached). Indeed, direct comparison with other reports in the literature is difficult as there is no standard metric for assessing enrollment success and benchmarking with other programs. We attempted to collect detailed data on recruitment but were limited by the willingness and resources of community partners to complete and/or provide the data/information. We did not want to overburden our community partners as recruitment was the only initial step and a continuing process in the early months. They were required to plan and deliver family programs soon after recruitment began.

It is clear that different recruitment strategies vary substantially in cost in terms of money and manpower per participant recruited. We are not able to provide the cost-effectiveness of recruitment strategies nor an investment yield ratio. Many of our strategies were resource consuming; however, some of these events (e.g., kick-off ceremony, promotion activities, and mobile counters) are likely to have a more sustained impact on community residents (e.g., raising awareness, normalize participation in family programs, and build social networks), which sets the stage for future recruitment in the community. We believe that these strategies were useful and successful in recruiting community residents. We also acknowledge that the practical constraints of financial and human resources often limit the extent of applying various recruitment strategies.

Recruitment is central to any research project, and recruitment itself should be well documented and researched (20). For our project, successful recruitment necessitated using more resource intensive and personalized approaches. Community-based recruitment efforts should involve members of the targeted community in the planning, preparation, and recruitment process. Recruiting the hard-to-reach should be a priority, and more resources are needed to do so and to evaluate more systematically. Further research is needed to examine the effectiveness of tailoring recruitment strategies to varying populations, especially for those conducted in the community.

## Ethics Statement

Ethics approval was obtained from the Institutional Review Board of the University of Hong Kong/Hospital Authority Hong Kong West Cluster.

## Author Contributions

The first author JC led the development of this manuscript. AW, KN, TL, and SC contributed to the design, implementation, and data collection of the project. TL and SS made continual input as the draft progressed and approved the final draft for submission. All authors read and approved the final manuscript.

## Conflict of Interest Statement

The authors declare that the research was conducted in the absence of any commercial or financial relationships that could be construed as a potential conflict of interest.

## Abbreviations

CFSC, Christian Family Service Centre.
